# Time-of-Day Effects in Resting-State Functional Magnetic Resonance Imaging: Changes in Effective Connectivity and Blood Oxygenation Level Dependent Signal

**DOI:** 10.1089/brain.2021.0129

**Published:** 2022-08-04

**Authors:** Liucija Vaisvilaite, Vetle Hushagen, Janne Grønli, Karsten Specht

**Affiliations:** ^1^ Department of Biological and Medical Psychology, University of Bergen, Bergen, Norway.; ^2^ Mohn Medical and Imaging Visualization Centre, Haukeland University Hospital, Bergen, Norway.; ^3^ Department of Radiology, Haukeland University Hospital, Bergen, Norway.; ^4^ Department of Education, UiT/The Arctic University of Norway, Tromsø, Norway.

**Keywords:** BOLD, DCM analyses, effective connectivity, resting state fMRI, time of day

## Abstract

**Introduction:**

In the light of the ongoing replication crisis in the field of neuroimaging, it is necessary to assess the possible exogenous and endogenous factors that may affect functional magnetic resonance imaging (fMRI). The current project investigated time-of-day effects in the spontaneous fluctuations (<0.1 Hz) of the blood oxygenation level dependent (BOLD) signal.

**Method:**

Using data from the human connectome project release S1200, cross-spectral density dynamic causal modeling (DCM) was used to analyze time-dependent effects on the hemodynamic response and effective connectivity parameters. The DCM analysis covered three networks, namely the default mode network, the central executive network, and the saliency network. Hierarchical group-parametric empirical Bayes (PEB) was used to test varying design-matrices against the time-of-day model.

**Results:**

Hierarchical group-PEB found no support for changes in effective connectivity, whereas the hemodynamic parameters exhibited a significant time-of-day dependent effect, indicating a diurnal vascular effect that might affect the measured BOLD signal in the absence of any diurnal variations of the underlying neuronal activations and effective connectivity.

**Conclusion:**

We conclude that these findings urge the need to account for the time of data acquisition in future MRI studies and suggest that time-of-day dependent metabolic variations contribute to reduced reliability in resting-state fMRI studies.

**Impact statement:**

The results from this study suggest that the circadian mechanism influences the blood oxygenation level dependent signal in resting-state functional magnetic resonance imaging (fMRI). The current study urges to record and report the time of fMRI scan acquisition in future research, as it may increase the replicability of findings. Both exploratory and clinical studies would benefit by incorporating this small change in fMRI protocol, which to date has been often overlooked.

## Introduction

During the past two decades, there has been an exponential increase in the number of publications related to brain functional connectivity (FC), as measured by functional magnetic resonance imaging (fMRI) (Pawela and Biswal, 2011). To date, research on FC has boomed, covering a variety of disciplines (Raichle, 2015), such as neurology (Fox and Greicius, 2010), psychiatry (Woodward and Cascio, 2015), and oncology (Bruno et al., 2012).

Resting-state FC (rs-FC) measures the temporal correlation of a spontaneous blood oxygenation level dependent (BOLD) signal among the different brain regions. Even though the rs-FC networks have been found to be stable across the population (Damoiseaux et al., 2006), the literature indicates vast individual differences based on a number of traits. Current evidence suggests that the changes or disruptions in the rs-FC may serve as a biomarker of brain diseases, such as dementia (Broyd et al., 2009) and other neurodegenerative diseases (Brier et al., 2012). Deficits in cognitive performance are associated with Alzheimer's disease (Zhou et al., 2013), mild cognitive impairment, autism spectrum disorders (Hull et al., 2016), and schizophrenia (Sheffield and Barch, 2016), all of which are also reflected in the rs-FC.

A recent publication on large-scale U.K. Biobank data reported that differences in cognitive performance in healthy individuals are associated with differences in the rs-FC, specifically the default mode network (DMN), where the neural associations are also shared with individual differences in educational attainment and household income (Shen et al., 2018). In addition, the stimulus-related BOLD responses in most brain areas are found to change throughout the course of the day, with a typical decrease from morning to evening hours (Marek et al., 2010).

The presence of diurnal brain dynamic is evident in the literature regarding attention, which urges the notion that psychological and neuropsychological assessments, together with work and school schedules, should instead be planned in accordance with circadian rhythmicity, age, and individual chronotype, rather than based on socioeconomic considerations, as the former are not easily adjusted (Valdez, 2019). Studies applying forced desynchrony protocol confirm that synchronized alternations between bursts of action potentials and periods of membrane hyperpolarization of cortical neurons are directly modulated by endogenous circadian rhythmicity (Lazar et al., 2015). Consequently, chronotype markedly influences time-of-day modulation on cerebral activity patterns, where studies suggest that larks and owls exhibit an inverted relationship curve throughout the day (Christie and McBrearty, 1979; Horne et al., 1980).

Notably, brain imaging studies using cognitive performance suggest that some but not all tasks vary throughout the day. For instance, insight-based problem performance is shown to increase at “non-optimal” time-of-day, contrary to the performance of solving analytical problems (Wieth and Zacks, 2011).

In spite of the endogenous nature of circadian rhythms in several brain functions, the time-of-day dependent variability in resting-state-fMRI (rs-fMRI) is not consistently reported. FC in the DMN is reported to exhibit a rhythmic pattern, with its peak in the morning and lower in the later hours of the afternoon (Blautzik et al., 2013). In addition, the variability in rs-FC has been reported in the medial temporal lobes (MTL) when comparing morning and evening scans (Shannon et al., 2013). The authors report that MTL exhibit stronger local connectivity in the morning, which shifts in the evening to distributed connections between MTL, frontal, and parietal regions (Shannon et al., 2013). When the magnitude of cerebral blood flow and FC in the DMN is examined, a consistent decrease in DMN FC, particularly in the posterior cingulate cortex (PCC) and the medial prefrontal cortex (mPFC), across the daytime is found (Hodkinson et al., 2014).

Recent reports do support the notion that FC in the DMN is affected by time-of-day and chronotype (Facer-Childs et al., 2019) and a steady decrease of global signal fluctuation and regional BOLD fluctuations together with whole-brain rs-FC throughout the day (Orban et al., 2020). Orban et al. (2020) further present evidence that the association between time-of-day and the reductions in global rs-FC is stronger than the association with fluid intelligence measure, which reflects the individual capacity for spot-on reasoning regardless of the previously acquired knowledge base. Given what has been cited earlier, it is evident that the diurnal variation in rs-FC networks is present.

Further evidence for the circadian rhythmicity effect on neural measures is put forward by studies investigating cortical excitability (Huber et al., 2012), white matter microstructures (Voldsbekk et al., 2020), brain volume (Karch et al., 2019; Nakamura et al., 2015), gray matter density, cortical surface, and thickness (Trefler et al., 2016). Nakamura et al. (2015) report that brain volume changes significantly across the day, with larger brain volumes in the morning compared with the evening. These changes in brain volume are observed across multiple populations, such as healthy elderly individuals, patients suffering from multiple sclerosis, mild cognitive impairment, and Alzheimer's disease (Nakamura et al., 2015). Morphometry measures suggest that increased volumes of cerebrospinal fluid (CSF) are associated with decreased volumes of gray and white matter (Trefler et al., 2016) and that the extracellular space volume is reduced in large parts of the white matter from morning to evening (Voldsbekk et al., 2020). It is suggested that the volume changes might also be associated with the level of individual hydration, where levels of hydration have previously been shown to have an effect on evoked BOLD signal captured by fMRI (Kempton et al., 2011).

Given the existing body of literature, it is reasonable to question whether the captured individual differences in a healthy population or group differences in clinical neuroimaging literature are, indeed, independent of the timing of the acquired scan (Karch et al., 2019; Trefler et al., 2016; Voldsbekk et al., 2020). It is not recognized as a common practice to report the image acquisition time in neuroimaging studies; therefore, it remains a possibly biasing factor. Exploring the effect of time-of-day in neuroimaging would contribute to a more robust reporting of data collection procedures, which would allow for easier replication of findings if found significant.

Given the body of literature pointing forward to the existing time-of-day effects in rs-FC, cortical excitability, white matter microstructures, brain volume, gray matter density, cortical surface, and thickness we aimed at exploring a novel way to analyze fMRI data to contribute and complement the existing findings. Dynamic causal modelling (DCM) is a recent approach to analyze fMRI data developed by Friston and Dolan (2010). DCM, contrary to mainstream fMRI analysis techniques, is a generative modeling technique that incorporates the Bayesian framework to estimate the nonlinear relationship between brain regions, attempting to explain the connectivity based on predicted hidden neural states (Friston et al., 2003b, 2014).

The application of DCM is not limited to the analysis of effective connectivity in task-related fMRI studies, but it has recently been extended to rs-FC, by parametrizing the spectral characteristics of the neuronal fluctuations (Friston et al., 2014; Razi et al., 2015). It can be applied with a predefined set of the region of interest (ROI) or as a purely data-driven approach (Friston et al., 2003a, 2014). Based on the rs-FC time-series, DCM generates measures of effective connectivity—that is, a directional relationship between the selected ROIs––and separately models the hemodynamic parameters (Balloon model), amplitude (α), and spectral density (β) (Friston et al., 2014). The motivation behind choosing this approach to address the time-of-day effects in the current work was twofold: First, the previous work so far did not address the vascular effects and neuronal parameters separately, and second, given the results from FC, use them as ROIs to investigate whether the effective connectivity would mimic the previously reported rs-FC results.

Given all what has been cited earlier, the aim of the current study was to investigate diurnal (time-of-day effect) change in resting-state effective connectivity, measured by DCM. For the scope of this study, three large-scale rs-FC networks were selected: the DMN, saliency network (SN), and Central Executive Network (CEN). The network effective connectivity was compared at six different timespans throughout the day (from 09:00 until 21:00). It was expected to observe the diurnal changes in neuronal activity and/or metabolic response (as generated by DCM).

## Methods

The Human Connectome Project data release “S1200” was used in the current study. For complete information about the dataset, please see Van Essen et al. (2013). The current study has been approved by the Regional Ethics Committee in Bergen, Norway (REK no. 31972 ReState).

### Participants

For the purposes of the current study, the participants scanned from 9:00 to 21:00 were selected from the complete dataset “S1200.” This decision was made given that only a few participants were scanned before 9:00 and after 21:00. The aim was to have an equal sample size for each timespan, with equal gender distribution for each timespan. The mid-scan time was used to allocate the participants to six groups of selected timespans (9:00–10:59, *N* = 96; 11:00–12:59, *N* = 100; 13:00–14:59, *N* = 100; 15:00–16:59, *N* = 100; 17:00–19:00, *N* = 100, 19:00–21:00, *N* = 98), in total 594 participants (310 females).

The Human connectome project (HCP) data were collected for them to reflect a naturally occurring diversity within the U.S. population; therefore, the inclusion was broadened to those who are smokers, overweight, and had a history of drinking or recreational drug use. All participants at the time of participation were healthy adults ranging from 22 to 35 years of age. The data were collected over 3 years on a single 3 Tesla (3T) scanner. The participant pool primarily consisted of subjects living in Missouri, in families with twins. Exclusion criteria were neurodegenerative disorders, documented neuropsychiatric disorders, neurological disorders, high blood pressure, and diabetes. For a complete list of inclusion criteria, please see Van Essen et al. (2013).

### Procedure

Participants were scanned at the Washington University in St. Louis over a 2-day and one-night visit. Informed consent was signed by the participants at the beginning of day 1. In accordance with previously run pilot studies, a consistent scanning schedule was maintained for all the participants in the study, unless a re-scan was required. For more details, see Van Essen et al. (2013). A mock scanning trial with feedback on the head motion was run before the first scanning. The data collection scanning for each participant was scheduled once a day with two rs-fMRI acquisitions each—one on day 1 (right/left and left/right phase encoding) and the other on day 2 (left/right and right/left phase encoding). The average time for each scanning occasion was 14.4 min, during which time the room was darkened. The participants were asked to lie still with their eyes open and fix their gaze on a bright fixation cross in the darkness; however, no eye-tracking method was used to ensure that the participant, indeed, maintained awake. During day 1 scanning, resting-state scanning was conducted after the structural MRI session and followed by the task-fMRI session. On day 2, the resting-state acquisition was conducted after a diffusion imaging scan followed by a task-fMRI session (Van Essen et al., 2013). The clock time for each scanning day varied from 7:00 to 22:00. Complete data collection procedures can be found in Van Essen et al. (2013). Given that the participants were scanned at different hours on day 1 and 2, we only used the scans from day 1 acquisition, resulting in a between-subject design.

### Image acquisition

rs-fMRI data collection was carried out in accordance with an optimized fMRI image acquisition protocol as determined by the HCP piloting. A custom Siemens 3T “Connectome Skyra” scanner was used to record the data for all participants. The scanner was equipped with a 32-channel head coil, custom gradient coils, and gradient power amplifiers boosting the gradient strength to 100 mT/m. rs-fMRI data were acquired in two sessions: first, 2 × 14.4 min runs, right/left and left/right phase encoding on day 1, and subsequently, 2 × 14.4 min runs left/right and right/left phase encoding on day 2, a total of 1-h rs-fMRI. A gradient-echo multiband EPI imaging sequence was used to acquire rs-fMRI data. rs-fMRI image acquisition settings were as follows: repetition time (TR) of 720 ms, echo time (TE) of 33.1 ms, 52° flip angle, the field of view 208 × 180 mm (readout × phase encoding), and slice thickness 2.0 mm; 72 slices; 2.0 mm isotropic voxels; and a multiband factor of 8 (for more information see Glasser et al., 2016; Uğurbil et al., 2013). In addition, high-resolution T1-weighted structural images were obtained with the following parameters: TR 2400 ms, TE 2.14 ms, inversion time (TI) 1000 ms, flip angle 8°, FOV 224 × 224 mm, and 0.7 mm isotropic voxels (Uğurbil et al., 2013).

### Image processing

For the purpose of this study, the data were acquired and preprocessed in accordance with the “Human Connectome Project minimal pre-processing pipeline” (please refer to Glasser et al., 2013 for details). Standard preprocessing steps—such as correcting for distortions and spatial alignment—were performed. In addition to the mentioned standard procedures, the data were also corrected for spatial distortions, aligned across modalities, and brought into a standard spatial atlas coordinate system (MNI space). The only variation from standard preprocessing procedures was due to the bore diameter of the scanner being 56 cm, which is smaller than the standard Siemens 3T Skyra size (70 cm diameter). The reduced diameter and lack of a customized patient table resulted in higher placement of the patient table in the bore and the participants' heads not being centered along the gradient isocenter, meaning that the scans have greater than normal gradient distortions, which have been corrected for in the HCP preprocessed data used in this project; for more details, see Van Essen et al. (2013).

### Analysis

To remove other nuisance confounds, the minimally preprocessed data were further processed before the DCM analysis. Using linear general models, as implemented in SPM12, the effects of the head movement (12 movement parameter) and the signal from white matter and CSF areas were regressed out from the time series. This was done by extracting the time course from a spheric volume with a radius of 6 mm at MNI coordinates ([0 − 24 − 33] and [0 − 40 − 5]), respectively. The time courses and the movement parameter were then incorporated in a general linear model, using SPM12, which formed the basis for extracting the time courses for the subsequent DCM analyses. This procedure has been performed separately for each of the two rs-fMRI sessions (left/right and right/left phase encoding, hereafter referred to as LR/RL) and for each individual separately.

There were eight regions of interest selected for the current study: four nodes in the DMN (mPFC; MNI coordinates: [3 54 − 2]; PCC [0 − 52 26]; right inferior parietal cortex [48 − 69 35]; left inferior parietal cortex [−50 − 63 32]), two nodes of SN (anterior insula [37 25 − 4]; anterior cingulate cortex [4 30 30]), and two nodes of CEN (dorsolateral prefrontal cortex [45 16 45]; posterior prefrontal cortex [54 − 50 50]). Each ROI was a sphere with a radius of 6 mm. Cross-spectral-density DCM (csdDCM) implemented in SPM 12.2 was used to extract time series for each ROI (Friston et al., 2014; Razi et al., 2015). Due to computational limits, only eight nodes could be processed. These particular eight nodes were selected based on the previous publications investigating circadian mechanisms in resting state (Blautzik et al., 2013; Facer-Childs et al., 2019; Shannon et al., 2013).

The effective connectivity was estimated for each participant and for the LR and RL sessions separately. The Parametric Empirical Bayes (PEB) framework was used to estimate the joint effective connectivity per timespan (Zeidman et al., 2019).

A hierarchical PEB procedure was applied to test varying design matrices against the time-of-day model. First, a PEB analysis was conducted for each timespan and for LR/RL separately. Thereafter, the corresponding 12 PEB results (6 timespans, 2 sessions) were subjected to a series of second-level PEB analyses, where 18 models for time-of-day effects were explored. The models were conjointly specified for LR/RL, such that the two sessions were finally averaged at this level of the analysis. The following models were specified:

(1) Model 1–6: the expectation of a *deviation from the mean for one single timespan*
(2) Model 7–11: the expectation of a *deviation from the mean over two adjacent timespans*
(3) Model 12–17: the expectation of *phase-shifted variants of a sinusoid function*—approximating a circadian rhythm, peaking at different timespans
(4) Model 18: The null model*:* the expectation of *no predictions at any time span*

These 18 models were defined as different design matrices, comprising one column for the overall mean, one column for the mean-corrected model as described earlier, and five columns indicating the different timespans and for parametrizing the repeated measurements, that is, combining the LR and RL sessions. The results were compared by using Bayesian model comparison (BMC). The winning model was explored at a cutoff of the posterior probability of pp >0.95. These analyses were conducted for the effective connectivity matrix (A-matrix, 8 × 8 parameter).

Then, the same procedure was repeated for the hemodynamic parameters of the Balloon model; *transit time*, *epsilon,* and *decay*, and the parameters α (reflecting amplitude) and β (reflecting the spectral density of the neural fluctuations). The parameter *transit time* was estimated for each of the eight ROIs, and *decay*, *epsilon,* α, and β are global parameters.

## Results

**FIG. 1.**
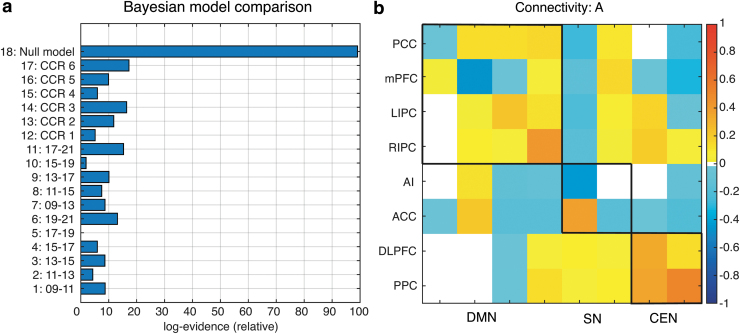
The figure summarizes effective connectivity results from the hierarchical Parametric Empirical Bayes analysis. **(a)** Bayesian Model Comparison on the connectivity parameter, where model 1–6 assumes deviating effect only for single timespans of 2 h, whereas model 7–11 assumes deviating effects for timespans of 4 h. The model 12–17 modeled different phase shifted version of an idealized circadian rhythm. Model 18 was the null model that assumed no time-of-day effects. **(b)** The estimation of effective connectivity (from columns to rows) across all subjects. The leading diagonal elements represent self-connections in logarithmic scale relative to the prior mean of −0.5 Hz. White space represents no significant effect at pp level >0.95.

The BMC of the time-of-day variations in the effective rs-FC showed that the highest model evidence (model accuracy minus model complexity) was the null model (Fig. 1a). The effective connectivity matrix of the null model with a posterior probability >0.95 (pp; the updated probability of the model being true after comparison with other models) is displayed in Figure 1b. In other words, the Bayesian model suggests that the neural activity in large-scale networks remains stable throughout the day.

**FIG. 2.**
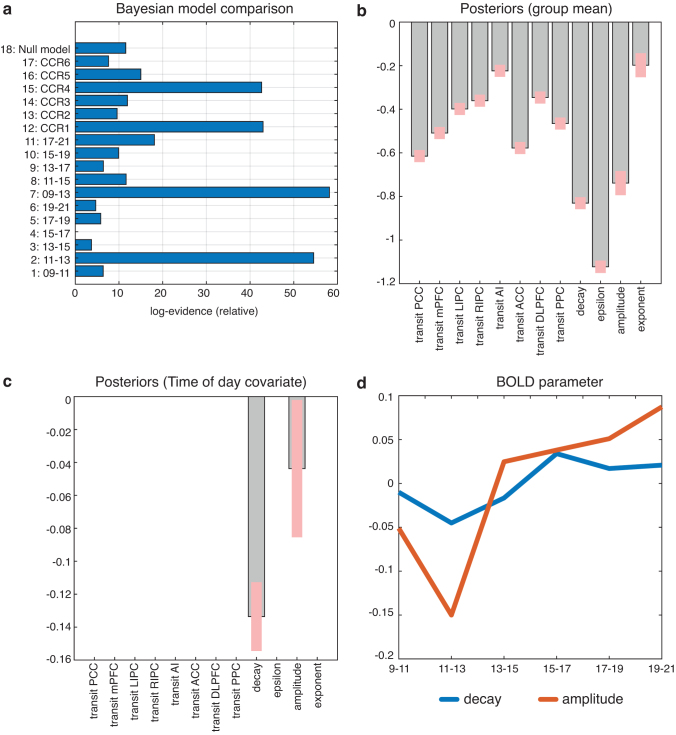
The figure summarizes hemodynamic parameter results from the hierarchical Parametric Empirical Bayes analysis. **(a)** Bayesian Model Comparison on the hemodynamic parameter *transit time*, *decay*, *epsilon*, as well as *Cross-spectral-density amplitude* and *Cross-spectral-density exponent.* **(b)** Group means for the posterior estimates of the hemodynamic parameter, displayed at a posterior probability of pp >0.95. **(c)** Posteriors of the winning model, displayed at pp >0.95. **(d)** Time course of the two significant posteriors decay and CSD amplitude after time-span-wise averaging with Bayesian Model Averaging. CSD, cross sprectral density.

Performing a model comparison on the hemodynamic parameters [*transit time*, *epsilon*, and *decay*, as well as amplitude (α) and spectral density (β)] BMC favored model 7, which predicted a deviation from the mean for the two timespans early in the morning, 09–13 (Fig. 2a). Moreover, three other models showed high model evidence, which were related variants of the winning model by predicting a deviation between 11 and 13 (model 2) or they were a sinusoid function peaking between 11 and 13 (model 12) or being the inverted waveform with a minimum between 11 and 13 (model 15). Group means relative to the fitted model are shown in Figure 2b. The posterior values seen in Figure 2c represent the level of parameter movement in accordance with the winning model (model 7) at the pp >0.95 level. The posterior values for the *decay* and the *amplitude* of the cross spectral density (CSD) function are significantly reduced for the timespan 9–13 (model 7). Finally, Figure 2d displays how the posterior values for decay and amplitude vary over the day after the respective DCMs were averaged for each timespan using Bayesian Model Averaging—in other words, the neural metabolic response measured by BOLD. Using the parameters for the winning model, the effect sizes for *decay* and CSD *amplitude* are *d* = 0.23 and *d* = 0.28, respectively.

## Discussion

To the best of our knowledge, this study is the first to investigate the time-of-day influence on resting-state neuronal activity (effective connectivity) and metabolic demand (BOLD signal). The main findings of the current study are that although the functional and effective connectivity of the brain remains stable throughout the day, the hemodynamic parameters defining the BOLD as generated by the DCM exhibit a variation between the selected timespans.

The effective connectivity in three large-scale resting-state networks, namely DMN, SN, and CEN, was found to remain stable across morning, noon, and evening hours (from 09:00 to 21:00). These findings are contradictory to previously reported results from FC, that is, a correlational relationship between brain regions, that indicate a shift in DMN and MTL FC from morning to evening (Blautzik et al., 2013; Shannon et al., 2013). Interestingly, a recent publication using the same subjects (HCP release S1200) reports a cumulative global signal decrease and whole-brain FC decrease throughout the day (Orban et al., 2020), which appear as inconsistent with present findings of constant FC in DMN, SN, and CEN. However, the conflicting findings between the current and previously reported results are likely rooted in different methodological approaches used. In the present study, the dynamic relationship between functionally connected nodes was assessed by using a generative model, namely Dynamic Causal Modeling, which analyzes the bidirectional effect that each network exhibits with itself and others. By contrast, previous publications reported the FC, namely the correlational relationship between the nodes of the network, which is based on the temporal correlations of the BOLD signals (Facer-Childs et al., 2019; Orban et al., 2020). One might speculate that those correlative approaches are potentially biased by systematic variations of the underlying BOLD signal, as demonstrated by the present study. The main finding of our results is that the parameters defining BOLD signal, that is, *decay* and *amplitude*, indeed show a time-of-day effect (model no. 7), and, hence, methods that are based on directly analyzing the amplitude of the BOLD signal may be affected by the time-of-day effects. These effects might be caused by, for example, diurnal variations in blood pressure (Millar-Craig et al., 1978), as has been recently shown by Sjuls and Specht (2021). The authors note that blood pressure and body mass index introduce variance in resting-state networks, most significantly in the DMN (Sjuls and Specht, 2021).

The BOLD signal, according to the current findings, varies depending on the time-of-day, in particular between morning and afternoon, regardless of the effective network activity. The hemodynamic parameter *decay* and the *amplitude* of the CSD function all exhibited a significant relationship with the time-of-day dependent model (model no. 7) (Fig. 2c). The *decay* parameter reflects the rate of signal elimination (Friston et al., 2000). A decrease in this parameter indicates an increased resting cerebral blood flow (rCBF) and a faster elimination of the signal and may also cause a larger BOLD undershoot. Accordingly, the BMC-selected model predicts a faster decaying BOLD signal before noon and, subsequently, a slower BOLD signal decay in the afternoon. As the plot over time indicates (Fig. 2d), this appears to be a dynamic effect over the daytime, and the inclusion of further timespans may show a cyclic effect. A similar effect can be seen when examining the *amplitude* of the cross-spectral-density function that also demonstrates lower amplitude in the morning than afternoon. In contrast to the *decay*, CSD *amplitude* showed a pronounced minimum for the 11–13 timespan. This different temporal evolvement during the morning does also explain why all four models that showed high model evidence in BMC (Fig. 2a) were rated similarly. Interestingly, there were no region-specific effects in the *transit times*, which indicate that this is a global vascular effect. One might also speculate whether these globally appearing effects in *decay* and CSD *amplitude* might also—at least to some extent—explain the observed time-of-day dependent fluctuations of the global signal as observed by Orban et al. (2020), using data from the HCP, as well. Generally speaking, it should be noted that the effect sizes are considerably large, and some methods that are currently used for the analysis of resting-state data might to a different degree be affected by the observed variation in the BOLD signal characteristic. Therefore, we think that the current findings should be re-examined in future studies using different methods.

The shift of *decay* is in line with the studies on resting state, where the connectivity appears higher in the morning compared with the evening (Hodkinson et al., 2014). The time-of-day dependency of the BOLD signal as observed in varied *decay* and *CSD amplitude* parameters is supported by previously published results on brain volume (Karch et al., 2019; Nakamura et al., 2015; Trefler et al., 2016). The increase in *decay*, as seen in the current study, occurs in the afternoon as well as the previously reported increase of CSF in Trefler et al. (2016). Given that CSF is vital for regulating the CBF, it is plausible to speculate that the reduction in the rCBF caused by a peak in the timespans before noon (9–11 until 11–13) is associated with the lower levels of CSF. During the first half of the daytime, a decrease in blood pressure, heart rate, and CBF(V) is observed around 11–13, whereas cortisol is still high. These daily changes in blood parameters provide support for the described change in the hemodynamic parameters. It is reasonable to observe a slower BOLD response with the reduced CBF, and the *decay* is likely dependent on vascular signaling and relaxation time (Friston et al., 2000). Alternatively, these changes in BOLD signal could have occurred because of diurnal variation in underlying neural activity, which has been shown to exhibit somewhat circadian rhythmicity in theta, alpha, and beta frequencies (Cummings et al., 2000).

The authors of former publications controlled for chronotype, sleep duration, and quality (Blautzik et al., 2013; Shannon et al., 2013). In contrast, the current work was carried out on open access data that did not include information on chronotype or sleep depth. Even though the database includes the information regarding “bedtime” and “get up time,” these are not considered to be accurate measures for the chronotype as they are driven by socioeconomic obligations—for example, work/school hours—hence not measuring the individual free-running sleep–wake cycles. A recently published work by Facer-Childs et al. (2019) suggests that there are significant differences in FC between early and late chronotypes in the DMN. These differences are somewhat in line with the early findings of Merrow et al. (2005) and Weitzman et al. (1971), suggesting that different chronotypes have distinct rhythmicity patterns throughout the day, where the postlunch dip and morning rise in cortisol levels might differ (Merrow et al., 2005; Weitzman et al., 1971). Taking into account these reported significant differences, it might be plausible that the reported effects from the current study could have been even stronger, had accounting for different phenotypical profiles been possible.

There are some limitations to the current work that could be addressed in future studies. First and foremost, the data obtained from the HCP, despite being high in quality, were not collected to address the connectivity differences across the time-of-day and did not account for chronotype differences. Second, the analysis method used in the present study (DCM) differentiates between the parameters defining the hemodynamic response (BOLD) and connectivity measures, but no additional parameters that have been previously shown to affect resting-state connectivity could be included, such as blood pressure, cerebral blood flow, and respiration (Facer-Childs et al., 2019; Specht, 2020). Further, the study relied on between-subject design, that is, different subjects were randomly assigned to the timespans, whereas to obtain a truly comprehensive understanding of the circadian rhythmicity, a repeated measure (within subject design) would be best, where the same participant would undergo scanning at each timespan (Karch et al., 2019). Even though the individuals in the HCP were scanned twice (on day 1 and on day 2), the hours of image acquisition and the scanning protocol itself for the same participant differed between days; therefore, while aiming to avoid the additional bias, we have limited the participant pool to only day 1. This decision only allowed for between-subject design, which is not optimal for exploring diurnal variations on an individual basis. Finally, in the present sample, the family structure was not accounted for, which might have decreased the significance of the results. It has been previously shown that circadian rhythmicity, the tendency for early and late chronotypes, is, in part, genetic; therefore, family structure should be accounted for, especially in cross-sectional samples (Lopez-Minguez et al., 2015).

The fact that the significant time-of-day effect only occurred in the hemodynamic and not in the connectivity parameters has a critical implication for past, current, and future research studies. The results from research on group differences, which did not counterbalance or did not account for the time of scanning, might have been, in part, detecting the time-of-day differences rather than the true group differences; for instance, when a group of participants is examined during morning hours (e.g., patients) and another group in the evening (e.g., control group). The use of dynamic causal modeling in future studies could aid in explaining the relationship between BOLD signal and FC. These future achievements would heavily contribute not only by bringing consensus in diurnal rhythmicity and resting-state connectivity research but also for an in-depth understanding of brain activity.

In summary, the results from the current study contribute to the limited body of literature on time-of-day changes in the brain. The findings suggest that even though there is an observed variability during the course of the day in the hemodynamic response, which is captured by fMRI, the effective connectivity remains stable. The changes in the hemodynamic response possibly reflect the influence of time-of-day effects on the metabolic and vascular system. This may indicate that the BOLD signal is more susceptible to exogenous parameters than the brain activity itself. These findings urge the need for further separation between the hemodynamic response and neural activity as reflected by FC since the relationship between the two might be more complex than previously thought and finally not stable throughout the day. Further, the time-of-day dependent variation of the metabolic basis of the fMRI signal might also partly explain the low reliability of fMRI studies, given that the effect size is rather large for an effect that is typically not accounted for.
